# Mobile Phone–Based Confidential Social Network Referrals for HIV Testing (CONSORT): Protocol for a Randomized Controlled Trial

**DOI:** 10.2196/55068

**Published:** 2024-05-30

**Authors:** Jan Ostermann, Bernard Njau, Marco van Zwetselaar, Thespina Yamanis, Leah McClimans, Rose Mwangi, Melkiory Beti, Amy Hobbie, Salomé-Joelle Gass, Tara Mtuy, Nathan Thielman

**Affiliations:** 1 Department of Health Services Policy & Management University of South Carolina Columbia, SC United States; 2 Kilimanjaro Christian Medical University College Moshi United Republic of Tanzania; 3 Zwets IT Harskamp Netherlands; 4 School of International Service American University Washington, DC United States; 5 Department of Philosophy University of South Carolina Columbia, SC United States; 6 Kilimanjaro Clinical Research Institute Moshi United Republic of Tanzania; 7 Center for Health Policy and Inequalities Research Duke University Durham, SC United States; 8 Department of Clinical Research London School of Hygiene and Tropical Medicine London United Kingdom; 9 Department of Medicine Duke University School of Medicine Durham, NC United States

**Keywords:** confidential referrals, HIV counseling and testing, mHealth, mobile health, social networks, stigma, sub-Saharan Africa, Tanzania

## Abstract

**Background:**

Critical to efforts to end the HIV epidemic is the identification of persons living with HIV who have yet to be diagnosed and engaged in care. Expanded HIV testing outreach efforts need to be both efficient and ambitious, targeting the social networks of persons living with HIV and those at above-average risk of undiagnosed HIV infection. The ubiquity of mobile phones across many high HIV prevalence settings has created opportunities to leverage mobile health (mHealth) technologies to engage social networks for HIV testing outreach, prevention, and treatment.

**Objective:**

The purpose of this study is to evaluate the acceptability and efficacy of a novel mHealth intervention, “Confidential Social Network Referrals for HIV Testing (CONSORT),” to nudge at-risk individuals to test for HIV using SMS text messages.

**Methods:**

We will conduct the CONSORT study in Moshi, Tanzania, the commercial center and administrative capital of the Kilimanjaro Region in northern Tanzania. After qualitative formative work and pilot testing, we will enroll 400 clients presenting for HIV counseling and testing and 200 persons living with HIV and receiving care at HIV care and treatment centers as “inviters” into a randomized controlled trial. Eligible participants will be aged 18 years or older and live, work, or regularly receive care in Moshi. We will randomize inviters into 1 of 2 study arms. All inviters will be asked to complete a survey of their HIV testing and risk behaviors and to think of social network contacts who would benefit from HIV testing. They will then be asked to whom they would prefer to extend an HIV testing invitation in the form of a physical invitation card. Arm 1 participants will also be given the opportunity to extend CONSORT invitations in the form of automated confidential SMS text messages to any of their social network contacts or “invitees.” Arm 2 participants will be offered physical invitation cards alone. The primary outcome will be counselor-documented uptake of HIV testing by invitees within 30 days of inviter enrollment. Secondary outcomes will include the acceptability of CONSORT among inviters, the number of new HIV diagnoses, and the HIV risk of invitees who present for testing.

**Results:**

Enrollment in the randomized controlled trial is expected to start in September 2024. The findings will be disseminated to stakeholders and published in peer-reviewed journals.

**Conclusions:**

If CONSORT is acceptable and effective for increasing the uptake of HIV testing, given the minimal costs of SMS text reminders and the potential for exponential but targeted growth using chain referrals, it may shift current practices for HIV testing programs in the area.

**Trial Registration:**

ClincalTrials.gov NCT05967208; https://clinicaltrials.gov/study/NCT05967208

**International Registered Report Identifier (IRRID):**

PRR1-10.2196/55068

## Introduction

### Background

The Joint United Nations Programme on HIV/AIDS (UNAIDS) set for 2030 the ambitious 95-95-95 target: diagnosing 95% of all persons living with HIV, initiating antiretroviral therapy for 95% of those diagnosed, and achieving viral suppression for 95% of those treated. Traditional testing approaches have linked countless persons living with HIV to treatment; however, the cost-effectiveness of these approaches for reaching incrementally harder-to-reach persons living with HIV is declining. The 2022 Tanzania Demographic and Health Survey and Malaria Indicator Survey (TDHS-MIS) found that only 79.6% of Tanzanian women and 64% of Tanzanian men had tested for HIV during their lifetime [1]. Furthermore, despite the recommendation that those in the general population who test negative retest annually, only 36.5% of women and 30.6% of men reported testing for HIV in the previous 12 months. Thus, novel approaches that efficiently reach at-risk individuals are urgently needed.

Social and sexual networks play a critical role in HIV transmission [2], testing decisions [3,4], linkage to care [5], and adherence [6]. However, numerous barriers, including HIV-related stigma, legal concerns, and the risk of unwanted serostatus disclosure, can impede HIV-related communication within social and sexual networks [7,8]. The ubiquity of mobile phones across many high HIV prevalence settings, including those in low- and middle-income countries (LMICs), has created opportunities to leverage mobile health (mHealth) technologies to engage social networks along the HIV care continuum for contact tracing, partner notification, clinic engagement, adherence reminders, and support for persons living with HIV [9-11]. The privacy and confidentiality afforded by novel applications of mHealth technologies can help address stigma and legal concerns, and broadly improve the uptake of HIV testing.

This study describes the protocol for a randomized controlled trial (RCT) to evaluate the acceptability and efficacy of mobile phone–based “nudges” in the form of “Confidential Social Network Referrals for HIV Testing” (CONSORT) to reach high-risk individuals and encourage them to test for HIV. The study will adapt and use an existing, highly versatile mobile phone–based appointment reminder and incentive system (mParis) [12-14]. mParis resides in Tanzania and can autonomously send large numbers of SMS text messages according to prespecified algorithms, making it a low-cost tool that can easily be adapted and scaled. In previous work, we explored the hypothetical acceptability and efficacy of CONSORT [15]. The survey results from this work suggested high feasibility and moderate acceptability of CONSORT*.* This study will explore the actual acceptability and efficacy of the intervention.

This study aligns with Tanzania’s 2017-22 “Health Sector HIV and AIDS Strategic Plan (HSHSP-IV),” which listed as its first challenge that HIV testing services need to be more efficient and ambitious [16]. Acknowledging the unfinished business from HSHSP-IV, the “Health Sector Strategic Plan July 2021–June 2026” highlights persistently low rates of HIV testing for some groups, particularly men and young people [17]. If CONSORT is shown to be acceptable and effective, confidential, digital, chain-referral methods could greatly improve the reach and cost-effectiveness of HIV testing efforts. While the CONSORT system will be developed and tested using SMS text messaging in a low-resource setting, the confidential chain-referral approach and the system’s open-source architecture may be extended to promote other health behaviors across varied social networks, app-based technologies, health conditions, and geographic settings.

### Study Aims and Hypothesis

This study aims to evaluate the acceptability and efficacy of automated confidential SMS text messaging–based HIV testing invitations as a means of “nudging” individuals to test for HIV. The overall study hypothesis is that an automated confidential referral system, developed and deployed in the Kilimanjaro Region of Tanzania, will be acceptable to both index participants (inviters) and their referrals (invitees), and it will be effective for increasing uptake of HIV testing. 

### The CONSORT Intervention

**Figure 1:**
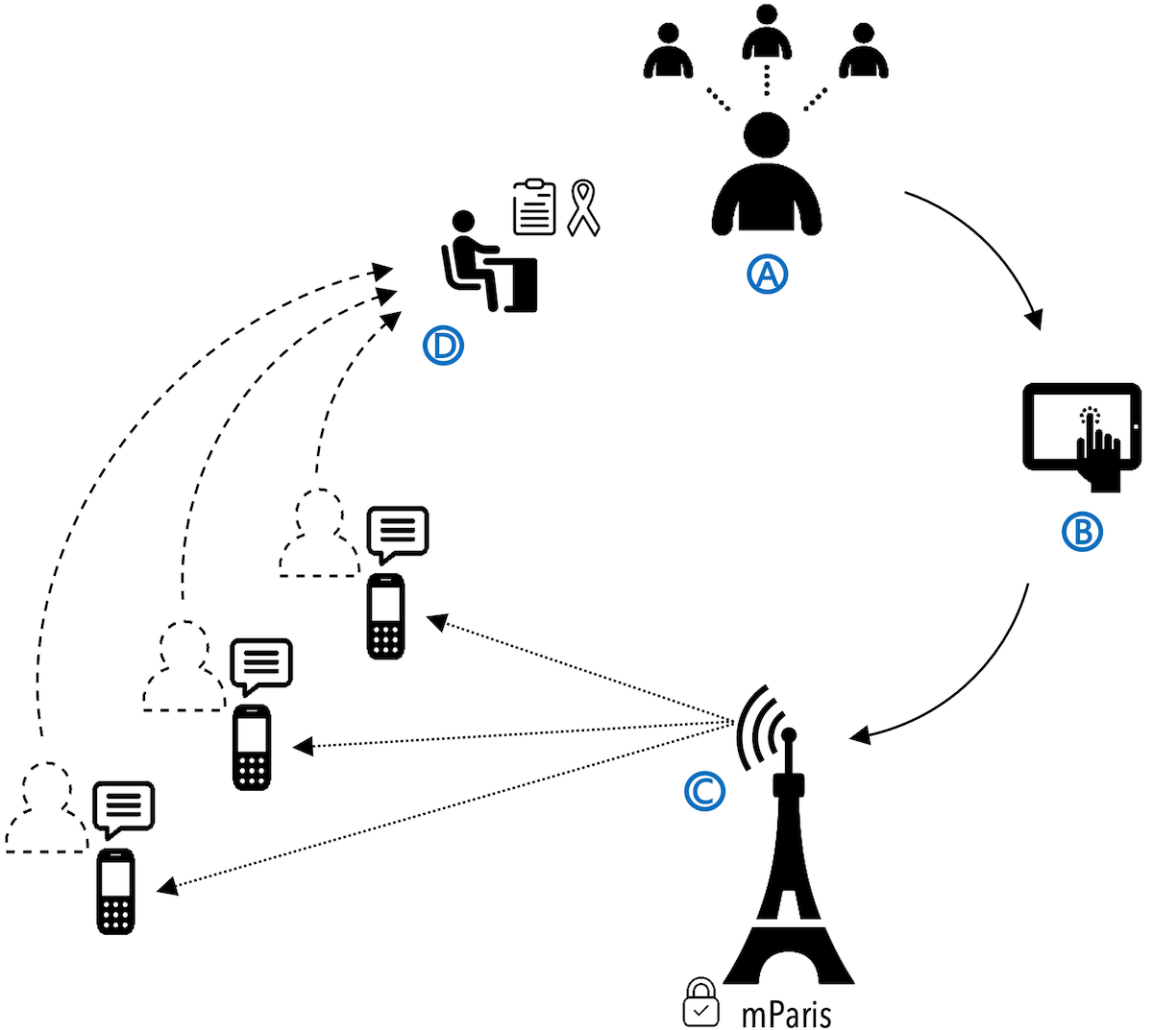
The Confidential Social Network Referrals for HIV Testing (CONSORT) process. (A) A consented “inviter” volunteers the phone numbers of social network contacts and (B) selects the invitation SMS text message. (C) A secure autonomous digital health system sends the selected SMS text messages to each “invitee” phone number. (D) An invitee responding to the invitation presents for HIV testing (“invitee tester”) and is offered the opportunity to become an inviter. mParis: mobile phone–based appointment reminder and incentive system.

The CONSORT process is shown in Figure 1. A consented inviter completes an HIV risk assessment and a survey of their social and sexual network contacts. Next, the inviter selects invitation messages from a menu of options to send to any of their network contacts who they think could benefit from HIV testing. Invitee phone numbers and SMS text messages are transferred securely to mParis. mParis autonomously sends the invitation SMS text message with a unique referral code to each invitee phone number. Invitees presenting for HIV testing with a referral code (invitee testers) will be offered the opportunity to become inviters (Figure 1).

## Methods

### Study Setting

The study will be conducted in Moshi, Tanzania. Moshi is the commercial center and administrative capital of the Kilimanjaro Region in northern Tanzania and has an estimated population of about 535,000 [18]. Moshi has 25 HIV counseling and testing (HCT) facilities that offer free HIV testing; many of these function as HIV care and treatment centers (CTCs), providing free HIV care to persons living with HIV [19]. HCT and CTC facilities with adequate volume to support the proposed study activities will be eligible to participate in the recruitment of inviters for the RCT. The uptake of HIV testing among invitees will be assessed across all HCT facilities in the study area.

### Study Sample

The RCT will include gender-balanced samples of 600 inviters, including 400 adult HCT clients and 200 adult persons living with HIV receiving care at participating CTCs.

### Inclusion and Exclusion Criteria

Eligible participants will be aged 18 or older and live, work, or regularly receive care in Moshi. Minors (<18 years) will be excluded, as it will not practically be possible to obtain assent to minors’ participation in a research study from a legal guardian.

### Recruitment

Clients presenting for HIV testing and persons living with HIV and receiving care at participating facilities will be approached consecutively for eligibility determination and informed consent and offered enrollment in the RCT. Recruitment will continue until the target sample sizes have been reached.

### Study Design

The study will evaluate the acceptability and efficacy of CONSORT*.* Because CONSORT may be a substitute for other referral options (eg, word-of-mouth referrals), full attribution of testing uptake to CONSORT would likely overstate the system’s effectiveness. To derive valid estimates of the effect of CONSORT, we will evaluate CONSORT in the context of a traceable substitute referral option: physical invitation cards.

#### Design of the RCT

**Figure 2:**
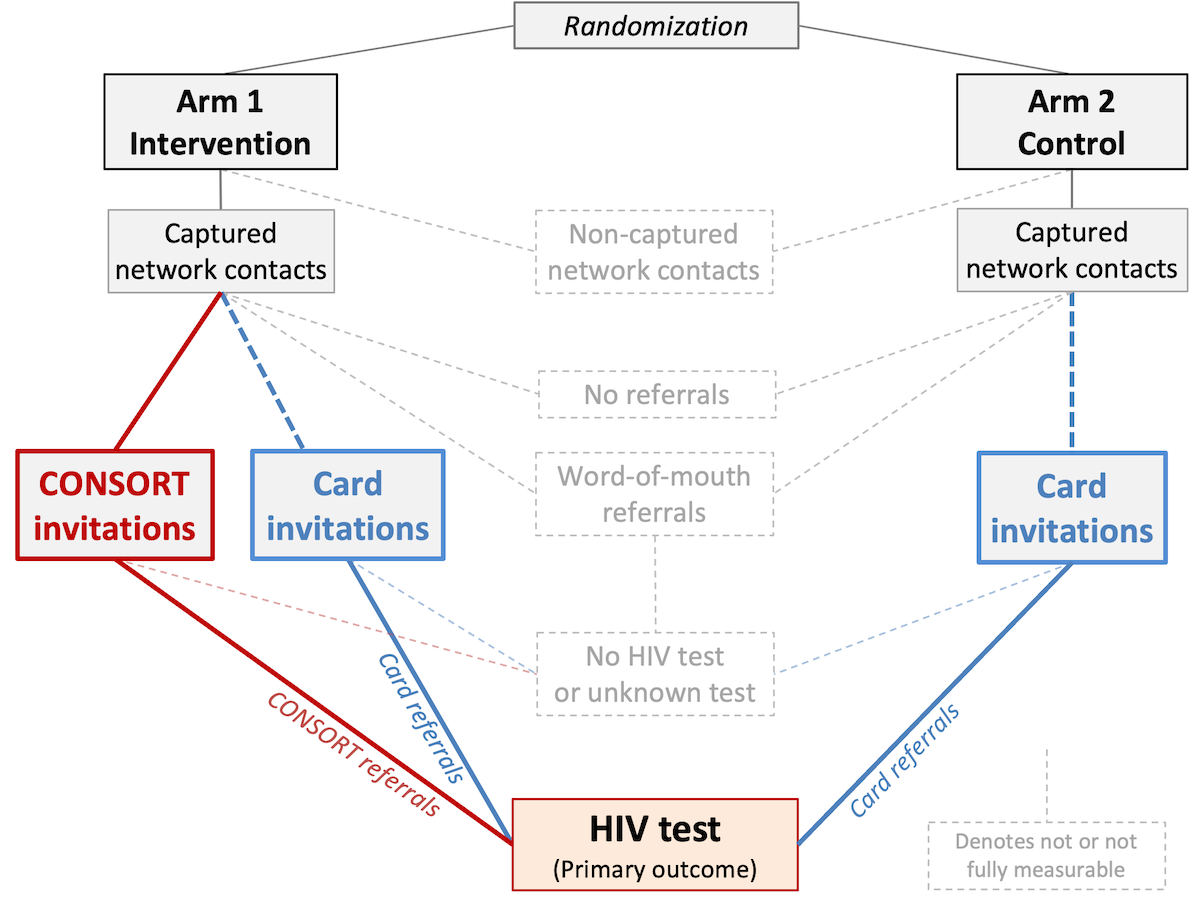
The design of the 2-arm randomized controlled trial (RCT). This RCT with 600 adult inviters will be conducted in Moshi, Tanzania. The primary outcome of interest is the uptake of HIV testing among invitees, defined as the number of invitees testing for HIV within 30 days of enrollment of the inviter per 100 inviters. While not all process elements can be observed, the RCT results yield an unbiased estimate of the efficacy of the Confidential Social Network Referrals for HIV Testing (CONSORT) for the primary outcome: inducing additional persons to test for HIV relative to physical invitation cards alone.

The design of the 2-arm RCT is shown in Figure 2. Arm 1 participants will be offered to extend CONSORT invitations or physical invitation cards to any of their network contacts. Arm 2 participants will be offered physical invitation cards alone. Arm 1 represents the intervention arm; Arm 2 represents the active control arm.

#### Assignment to Study Arms

Participant IDs will be randomly assigned to either arm 1 or arm 2 using a random number generator. The random assignment is expected to result in approximately equal numbers of participants in each study arm.

#### Blinding

Neither participants nor research staff will be blinded with respect to inviters’ study arm assignments.

### Primary Outcome

The primary outcome is counselor-documented uptake of HIV testing among invitees within 30 days of the enrollment of the inviter. For invitees who present for HIV testing (invitee testers) after a CONSORT invitation, referral codes, alphanumerical codes that are unique to the phone number of the invitee, will be documented by counselors in logbooks; for invitees presenting with physical invitation cards, counselors will collect the invitation cards.

### Secondary Outcomes

Secondary outcomes include the acceptability of CONSORT among inviters, the number of new HIV diagnoses, and the HIV risk among invitee testers. The assessments of secondary outcomes are detailed later on in their own section.

### Study Activities

#### Enrollment Survey

Participants will complete an interviewer-administered enrollment survey to assess their HIV serostatus, HIV-related behaviors (eg, HIV prevention behaviors, HIV testing history, number of partners, and concurrency) [20], and stigma [21-23], as well as key demographic, socioeconomic, and household characteristics that may correlate with the acceptability and efficacy of CONSORT [24,25]. Surveys will be conducted in Kiswahili. The data will be captured electronically using tablet devices.

#### Social Network–Based HIV Testing Invitations

The enrollment survey will include a survey of inviters’ preferences for extending HIV testing invitations within their social networks. Participants will be asked to think of social network contacts across multiple network dimensions, including partners, family members, friends, coworkers, and other people who are aged 18 years or older and who would benefit from HIV testing. Participants will be asked to whom they would prefer to extend an HIV testing invitation through a physical invitation card; arm 1 participants will also be able to select CONSORT invitations. At the end of the survey, inviters will be given the respective number of coded invitation cards, and arm 1 inviters will be able to send CONSORT invitations to their network contacts (Figure 1).

#### Phone-Based Follow-Up Survey

The extent to which physical invitation cards were distributed to invitees will be ascertained through self-reports from inviters during a phone-based follow-up survey after 30 days.

#### Assessment of Primary Outcome

The primary outcome measure is counselor-documented uptake of HIV testing by invitees within 30 days of the enrollment of the inviter. All clients presenting for HIV testing will be asked if they received an HIV testing invitation by SMS text message or a physical invitation card. Counselors will document referral codes from SMS text messages and collect invitation cards. Referral codes will be validated against a database of referral codes issued. A match is interpreted as an invitee presenting for HIV testing.

#### Assessment of Secondary Outcomes

The acceptability of CONSORT among inviters will be described by the percentage of inviters extending at least 1 CONSORT invitation and the average number of CONSORT invitees per inviter. This outcome is assessed only for arm 1 participants.

For invitee testers, basic sociodemographic and risk characteristics (age, gender, marital status, pregnancy status, and previous testing), as well as their HIV test result, will be abstracted in aggregate form from administrative data collected for reports to Tanzania’s National AIDS Control Programme (NACP). Clients testing positive for HIV will be linked to care at a local CTC, following NACP guidelines [16]. Invitee testers consenting to become inviters will complete the same enrollment survey as their inviters, which includes a comprehensive HIV risk assessment.

### Participant Retention

Inviters who choose to extend physical invitation cards to their network contacts will be recontacted by phone after 30 days and asked whether they distributed these cards to their contacts. For all other inviters, study activities will end after the initial visit.

### Study Timeline

Details of the intervention will be finalized after formative qualitative work, including focus group discussions and in-depth interviews with HCT clients, CTC patients, and providers. Focus group discussions and in-depth interviews will explore ethical considerations and elucidate key client-side characteristics of CONSORT, including refining appropriate SMS text message content for invitees, defining parameters for the timing of invitation messages, and exploring the feasibility of incentives for inviters and invitees. Before the implementation of the RCT, the intervention will be pilot-tested with 50 adult HCT clients and 50 adult CTC patients. Formative work and pilot testing are expected to last 2 years.

Following formative work and a successful pilot test, the RCT is planned to commence in year 3 of this study. Enrollment in the RCT will continue until the target numbers of 400 HCT inviters and 200 CTC inviters have been reached. Assessments of invitation card distribution and HIV testing uptake will continue until 30 days after the last CONSORT invitation was sent through mParis and the last invitation card was issued.

### Statistical Analysis

#### Analysis of the Primary Outcome

**Figure 3:**
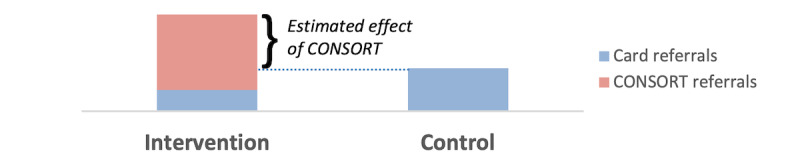
Visual representation of the estimated effect of Confidential Social Network Referrals for HIV Testing (CONSORT) on the primary outcome: number of invitees testing for HIV within 30 days per 100 inviters (hypothetical). The 2-arm randomized controlled trial with 600 adult inviters will be conducted in Moshi, Tanzania.

Efficacy will be analyzed descriptively by comparing, between the 2 study arms, the number of invitees testing for HIV within 1 month per 100 inviters (Figure 3). Analytically, efficacy will be modeled at the level of the inviter, using a zero-inflated negative binomial model, with the number of invitees returning for testing as the dependent variable. The primary explanatory variable will be a binary indicator variable for the study arm. Covariates will characterize variation in invitees’ testing uptake with inviters’ sociodemographic and network characteristics, HIV risk, and stigma.

#### Analyses of Secondary Outcomes

##### Acceptability Among Inviters

Logistic regression will be used to model inviters’ decisions to send at least 1 CONSORT invitation. Zero-inflated negative binomial models will model the number of invitations sent as a function of the covariates described above.

##### New HIV Diagnoses

Differences between study arms in the number of new HIV diagnoses among invitees, per 100 inviters, will be analyzed descriptively using a Fisher exact test.

##### HIV Risk Among Invitees

Differences in invitees’ sociodemographic and risk characteristics between study arms will be assessed descriptively using 2-tailed Student *t* tests and chi-square statistics.

#### Analytic Considerations

The analysis of the primary outcome will be stratified by cohort (HCT inviters: n=400; CTC inviters: n=200); generalized Hausman tests will evaluate whether the data can be pooled.

#### Sensitivity Analyses

Sensitivity analyses will assess the potential impact of missing data on estimates and describe variation in the acceptability and efficacy of CONSORT by cohort (HCT vs CTC inviters) and gender (male vs female inviters).

#### Statistical Power

The primary outcome analysis will compare rates of HIV testing among invitees across study arms (Figure 3). As a priori estimates of invitees’ testing rates in the control arm are not known, power calculations for differences in rates between study arms would be speculative. Instead, the power calculations refer to changes in the number of invitees presenting for testing, N_T_, which can be assumed to follow a Poisson distribution with a SE of sqrt (N_T_). Assuming that 20 (alternatively 40, 60, 80, or 100) invitees of arm 2 inviters present for testing, the study has 90% power to detect CONSORT-related differences of 7 (10, 13, 15, or 16) testers between study arms and 80% power to detect differences of 6 (8, 10, 11, or 13) testers between study arms. These calculations apply to cohort- or gender-specific as well as aggregate analyses.

### Reporting

This manuscript was prepared in accordance with the SPIRIT (Standard Protocol Items: Recommendations for Interventional Trials) checklist for clinical trial protocols. The completed checklist and Figure S1 can be found in Multimedia Appendices 1 and 2.

### Monitoring and Quality Assurance

Adherence to intervention protocols and the completeness and quality of study data will be monitored by the principal investigators or a designated study monitor. Electronic data capture on tablet devices and daily uploads to secure servers allow for the continuous monitoring of study activities in near real-time. All paper documents will be scanned. Rigorous quality assurance and quality control procedures will be established, including interviewer observation, validation and range checks during data entry, verification of entered data, and the monitoring of time stamps for electronic surveys.

### Patient and Public Involvement

Formative, qualitative work with HCT clients, CTC patients, and providers will elucidate key characteristics of CONSORT, including the selection of relevant social network contacts, appropriate message content for invitees, and defining parameters for the timing of invitation SMS text messages. Discussions will also focus on ethical considerations and the potential role of economic incentives as a means of motivating inviters to invite additional network contacts to test for HIV. Findings will be used to identify features of the CONSORT system that will maximize its acceptability among inviters and invitees.

### Ethical Considerations

This protocol was registered at ClinicalTrials.gov (NCT05967208) on July 25, 2023. The protocol was approved by the University of South Carolina Institutional Review Board (Pro00120208) in the United States; the Ethics Review Committee at Kilimanjaro Christian Medical University College (Protocol #1404); and the Tanzania National Institute for Medical Research (NIMR/HQ/R.8a/Vol.IX/4373). The project will consistently apply relevant ethical principles when working with individuals at risk for or affected by HIV. Protocol amendments will be submitted to these entities as required.

Informed consent documents will be developed in English and translated into Kiswahili. Trained research assistants will inform eligible individuals about the study’s purpose, procedures, risks, and benefits before obtaining informed consent for participation. The potential risks and benefits of research participation will be carefully explained in culturally appropriate and understandable language during the consent process. Consenting individuals will be enrolled in the study, with the study ID dictating their assignment to 1 of the 2 RCT study arms.

The data will be kept in compliance with relevant privacy regulations in Tanzania and the United States. Access to identifying information will be strictly limited. Study personnel will be instructed to keep the identities of all research participants confidential and will sign confidentiality agreements. The CONSORT system will be designed with a focus on confidentiality. Only encrypted versions of the invitee phone numbers will be stored in mParis, and information about the invitee will be stored separately from information about the inviter. Encrypted invitee phone numbers will be decrypted only as necessary for SMS scheduling and when "in flight" to the SMS text message center.

Inviters and their invitee testers who agree to participate in the research will receive between TSH 5000-10,000 (approximately US $2.20-US $4.40) as compensation for their time.

## Results

After qualitative formative work and pilot testing, enrollment in the RCT is expected to start in September 2024.

Research results from the CONSORT study will be disseminated through peer-reviewed publications, at national and international conferences, and through media outlets. To comply with the publication policies of the National Institute for Medical Research (NIMR) in Tanzania, approval for publications will be obtained from NIMR.

As many stakeholders and policy makers interested in this research reside in Tanzania, efforts will be made to translate the research abstracts into Kiswahili to make them more widely accessible. All system components and expertise needed to sustain and scale up CONSORT will be in place locally at the end of this project.

## Discussion

### Overview

This study will evaluate the acceptability and efficacy of automated, confidential SMS text message–based HIV testing invitations as a means of nudging individuals to test for HIV. If testing rates are higher in arm 1 invitees than arm 2 invitees, the results will support our hypothesis that an automated, confidential referral system is acceptable to both index participants (inviters) and referrals (invitees) and is effective for increasing uptake of HIV testing. If CONSORT is shown to be acceptable and effective, then confidential, digital, chain-referral methods could be used to improve the reach and cost-effectiveness of HIV testing efforts.

CONSORT’s potential is based on 3 key premises.

The first premise is that testing network contacts of persons living with HIV and those testing for HIV is an effective means for HIV case-finding. Identifying, tracing, and testing sexual contacts of persons with a sexually transmitted infection has been an essential component of public health sexually transmitted infection management for decades. Assisted HIV partner notification, by which health care workers elicit information about an index case's sexual partners and contact partners to request HIV testing, has been shown to be effective for increasing testing uptake and identifying new HIV infections [26-36]. A meta-analysis of 3 individual randomized trials [33-35] found that assisted partner notification, compared with passive referral, resulted in a 1.5-fold increase in HIV testing uptake. Moreover, the proportion of HIV-positive partners identified was 1.5 times higher with this approach [37]. Previous research also suggests that persons presenting for HIV testing have higher rates of HIV infection and higher rates of HIV risk behaviors than the general population [38,39]. Owing to similarities between members of social networks, a property known as homophily, referrals of HCT and CTC clients are, thus, also more likely to be infected with HIV than the general population. CONSORT*,* therefore, is well positioned to reach populations with an above-average risk of HIV infection and nudge them to test for HIV.

The second premise is that an assurance of confidentiality promotes referrals for HIV testing within social networks. CONSORT is conceptually related to confidential partner notification strategies [40,41], which are known to be highly acceptable to participants [42,43]. The evidence for technology-based notification systems for testing for sexually transmitted diseases, mainly derived from internet-based applications in high-income settings, is mixed: some show success [44-51], while others do not [41,52,53]. The efficacy of an impersonal, confidential approach to accessing network contacts in LMICs has yet to be evaluated. On the one hand, this approach may reduce the cost of contacting partners [54] and circumvent barriers such as nonreciprocal relationships [55], stigma [56], disclosure, and legal risks [57]. On the other hand, a confidential SMS text message is likely to be less motivating than personal communication. This study will compare the acceptability and efficacy of CONSORT versus other means of inviting social network contacts to test for HIV.

The third premise is that SMS text message–based nudges are inexpensive, effective tools for influencing health behaviors. Apart from incentives, reminders are among the simplest available nudging tools [58]. SMS text message–based nudges in the context of HIV testing remain rare, despite their low cost and nearly universal reach. While there is evidence that SMS text message–based interventions are feasible and acceptable in LMICs [59-63], and that nudging can influence health-related behaviors [64-66], relatively few studies have evaluated the use of SMS text messaging to encourage HIV testing [9,67-75]. We identified only 5 protocols that evaluated SMS text messaging for increasing uptake of facility-based HIV testing among at-risk adults in sub-Saharan Africa [9,70-72,75]. Although these studies demonstrated the strong potential of SMS text message–based messages to influence testing uptake, each has significant limitations, including using self-reports of HIV testing [67,70-72,75], using a combined intervention that included SMS text messaging with phone calls and in-person reminders [9], and limiting the intervention to young adults [9,72,74], women [72], or specific high-risk populations [71,75-82]. The design of this study is more rigorous in that (1) we will isolate the effect of SMS text messaging alone, and (2) actual testing uptake will be assessed in real time (rather than by self-report). Finally, results from CONSORT will be more broadly applicable, as the index (inviter) population will include participants from both sexes and across a greater range of ages, and it will extend beyond specific high-risk populations. With low costs of SMS text messages in the study area and an open-source and largely autonomous implementation, CONSORT may overcome the limits to growth encountered in traditional chain referral approaches [56] and support continuous, sustainable growth.

### Limitations and Considerations

This study is subject to several limitations and considerations.

First, feasibility considerations limit the study area to include only HCT facilities in Moshi municipality. Invitees may test outside the study area and may thus not be captured by our study. Furthermore, while referral codes and coded invitation cards collected from participating HCT providers offer definitive evidence of a completed HIV test, participants may test without disclosing receipt of CONSORT messages or invitation cards.

Second, the estimated effect sizes are not generalizable to other index populations, other areas in Tanzania, or other parts of Africa. While high mobile phone use rates and stable cellular network coverage suggest good technical feasibility of CONSORT, illiteracy, lack of trust in confidentiality assurances, and stigma remain potential challenges. In the formative work and the pilot study, we will explore options such as computer-assisted self-interviewing for capturing referral information, incentives, and system-level adjustments to maximize acceptability and efficacy.

Third, SMS text messages must be short and concise, can only contain text, and are not encrypted in transit. As implemented in this study, messages are presented as 1-way communication (although a phone number is provided if the invitee has questions). If successful, future work will explore the use of alternative communication options that allow for the secure transfer of audiovisual information and a more interactive experience (eg, through chatbots).

Finally, we note that this study is subject to 2 important ethical considerations: (1) inviters provide the phone numbers of their network contacts without the contacts’ consent, and (2) CONSORT invitees need to be informed that the SMS text messages they receive are part of a research project. Regarding the first consideration, Tanzania’s 2019 “National Comprehensive Guidelines on HIV Testing Services” sets a precedent in its section on Index Client Testing and Partner Notification [83]. The guidance outlines several assisted voluntary approaches to disclosing HIV status to the partners of index clients. One of the suggested approaches allows an HIV testing provider to contact the index client’s partners directly and confidentially for testing. Providers need the index client’s consent but not their partners’ consent. While this approach is primarily focused on index clients who are diagnosed with HIV, the guidelines emphasize "enhanced use of this approach throughout the country as among the new innovations to rapidly increase the number of PLHIV diagnosed.” Regarding the second consideration, SMS text messages to invitee phone numbers will include a statement indicating that the message is part of a research study. Throughout the study, we will continue discussions with the ethicists on our team to ensure that all procedures minimize potential risk of harm to both inviters and invitees, preserve strict confidentiality, and avoid potential stigmatization.

### Conclusion

In conclusion, the CONSORT approach, which combines the ubiquity of mobile phones with an assurance of confidentiality, holds promise for efficiently engaging higher-risk populations by nudging their network contacts to test [84-86]. If CONSORT is acceptable and effective for increasing uptake of HIV testing, it can be readily sustained and scaled, and it has the potential to shift current practices in HIV testing programs in the study area. Given the minimal costs of sending SMS text message reminders and the potential for exponential, but targeted, growth using chain referrals, this system could prove to be a cost-effective tool for accelerating Tanzania’s goal to reach HIV epidemic control by 2030. Leveraging social networks and technologies for nudging is readily extensible to other areas of public health, particularly where health concerns overlap and cluster within stigmatized or hard-to-reach social networks.

## Appendix

Multimedia Appendix 1 SPIRIT (Standard Protocol Items: Recommendations for Interventional Trials) figure depicting schedule of enrollment, interventions, and assessments.

Multimedia Appendix 2 SPIRIT (Standard Protocol Items: Recommendations for Interventional Trials) 2013 checklist.

Multimedia Appendix 3 Peer review report from the National Institute of Health.

## Abbreviations

CONSORT: Confidential Social Network Referrals for HIV Testing
CTC: HIV care and treatment center
HCT: HIV counseling and testing
HSHSP-IV: Health Sector HIV and AIDS Strategic Plan
LMIC: low- and middle-income country
mHealth: mobile health
mParis: mobile phone–based appointment reminder and incentive system
NACP: National AIDS Control Programme
NIMR: National Institute for Medical Research
RCT: randomized controlled trial
TDHS-MIS: Tanzania Demographic and Health Survey and Malaria Indicator Survey
UNAIDS: Joint United Nations Programme on HIV/AIDS

## Data Availability

The data sets generated or analyzed during this study are available from the corresponding author upon reasonable request.

## References

[1] (2023). 2023 Tanzania Demographic and Health Survey and Malaria Indicator Survey 2022: key indicators. The DHS Program.

[2] Klovdahl AS, Potterat JJ, Woodhouse DE, Muth JB, Muth SQ, Darrow WW (1994). Social networks and infectious disease: the Colorado Springs Study. Soc Sci Med.

[3] Huang ZJ, He N, Nehl EJ, Zheng T, Smith BD, Zhang J, McNabb S, Wong FY (2012). Social network and other correlates of HIV testing: findings from male sex workers and other MSM in Shanghai, China. AIDS Behav.

[4] Halkitis PN, Kupprat SA, McCree DH, Simons SM, Jabouin R, Hampton MC, Gillen S (2011). Evaluation of the relative effectiveness of three HIV testing strategies targeting African American men who have sex with men (MSM) in New York City. Ann Behav Med.

[5] Fuqua V, Chen YH, Packer T, Dowling T, Ick TO, Nguyen B, Colfax GN, Raymond HF (2012). Using social networks to reach Black MSM for HIV testing and linkage to care. AIDS Behav.

[6] Ghosh D, Krishnan A, Gibson B, Brown SE, Latkin CA, Altice FL (2017). Social network strategies to address HIV prevention and treatment continuum of care among at-risk and HIV-infected substance users: a systematic scoping review. AIDS Behav.

[7] Colombini M, Mutemwa R, Kivunaga J, Moore LS, Mayhew SH, Integra Initiative (2014). Experiences of stigma among women living with HIV attending sexual and reproductive health services in Kenya: a qualitative study. BMC Health Serv Res.

[8] Pérez GM, Hwang B, Bygrave H, Venables E (2015). Designing text-messaging (SMS) in HIV programs: ethics-framed recommendations from the field. Pan Afr Med J.

[9] Mugo PM, Wahome EW, Gichuru EN, Mwashigadi GM, Thiong'o AN, Prins HAB, de Wit TFR, Graham SM, Sanders EJ (2016). Effect of text message, phone call, and in-person appointment reminders on uptake of repeat HIV testing among outpatients screened for acute HIV infection in Kenya: a randomized controlled trial. PLoS One.

[10] Lester RT, Ritvo P, Mills EJ, Kariri A, Karanja S, Chung MH, Jack W, Habyarimana J, Sadatsafavi M, Najafzadeh M, Marra CA, Estambale B, Ngugi E, Ball TB, Thabane L, Gelmon LJ, Kimani J, Ackers M, Plummer FA (2010). Effects of a mobile phone short message service on antiretroviral treatment adherence in Kenya (WelTel Kenya1): a randomised trial. Lancet.

[11] Thakkar J, Kurup R, Laba TL, Santo K, Thiagalingam A, Rodgers A, Woodward M, Redfern J, Chow CK (2016). Mobile telephone text messaging for medication adherence in chronic disease: a meta-analysis. JAMA Intern Med.

[12] Ostermann J, Vasudevan L, Van Zwetselaar M, Moses S, Engadaya E, Mfinanga S (2018). Mobile Phone Assisted Reminder and Incentive System (mParis). Integrating mHealth reminders and conditional cash transfers to improve the timeliness of vaccinations in Tanzania [poster].

[13] Ostermann J, Vasudevan L, Van Zwetselaar M, Moses S, Engadaya E, Mfinanga S (2018). SMS reminders and conditional financial transfers to improve the timeliness of vaccinations in Tanzania. A pilot test of our Mobile Phone Assisted Reminder and Incentive System (mParis) [poster].

[14] Ostermann J, Vasudevan L, Baumgartner JN, Ngadaya E, Mfinanga SG (2019). Do mobile phone-based reminders and conditional financial transfers improve the timeliness of childhood vaccinations in Tanzania? Study protocol for a quasi-randomized controlled trial. Trials.

[15] Ostermann J, Njau B, Masaki M, Mtuy T, Itemba D, Hobbie A, Yelverton V, Moore S, Yamanis T, Thielman NM (2022). Feasibility, acceptability, and potential cost-effectiveness of a novel mobile phone intervention to promote human immunodeficiency virus testing within social networks in Tanzania. Sex Transm Dis.

[16] National AIDS control programme. Health Sector HIV and AIDS Strategic Plan (HSHSP IV) 2017-2022. Ministry of Health, Community Development, Gender, Elderly and Children.

[17] Health Sector Strategic Plan july 2021—june 2026 (HSSP V). Ministry of Health, Community Development, Gender, Elderly and Children.

[18] 2022 Tanzania Population and Housing Census. United Republic of Tanzania and National Bureau of Statistics.

[19] Ostermann J, Whetten K, Reddy E, Pence B, Weinhold A, Itemba D, Maro V, Mosille E, Thielman N (2014). Treatment retention and care transitions during and after the scale-up of HIV care and treatment in Northern Tanzania. AIDS Care.

[20] Ostermann J, Njau B, Mtuy T, Brown DS, Mühlbacher A, Thielman N (2015). One size does not fit all: HIV testing preferences differ among high-risk groups in Northern Tanzania. AIDS Care.

[21] Pantelic M, Boyes M, Cluver L, Thabeng M (2018). 'They say HIV is a punishment from god or from ancestors': cross-cultural adaptation and psychometric assessment of an HIV stigma scale for South African adolescents living with HIV (ALHIV-SS). Child Indic Res.

[22] Takada S, Nyakato V, Nishi A, O'Malley AJ, Kakuhikire B, Perkins JM, Bangsberg DR, Christakis NA, Tsai AC (2019). The social network context of HIV stigma: population-based, sociocentric network study in rural Uganda. Soc Sci Med.

[23] Kalichman SC, Simbayi LC, Jooste S, Toefy Y, Cain D, Cherry C, Kagee A (2005). Development of a brief scale to measure AIDS-related stigma in South Africa. AIDS Behav.

[24] Morris M, Morris M (2004). Overview of network survey designs. Network Epidemiology: A Handbook for Survey Design and Data Collection.

[25] Morris M, Wawer MJ, Podhisita C, Sewankambo N, Morris M (2004). The Thailand and Ugandan sexual network studies. Network Epidemiology. A Handbook for Survey Design and Data Collection.

[26] Myers RS, Feldacker C, Cesár F, Paredes Z, Augusto G, Muluana C, Citao S, Mboa-Ferrao C, Karajeanes E, Golden MR (2016). Acceptability and effectiveness of assisted human immunodeficiency virus partner services in Mozambique: results from a pilot program in a public, urban clinic. Sex Transm Dis.

[27] Henley C, Forgwei G, Welty T, Golden M, Adimora A, Shields R, Muffih PT (2013). Scale-up and case-finding effectiveness of an HIV partner services program in Cameroon: an innovative HIV prevention intervention for developing countries. Sex Transm Dis.

[28] de Olalla PG, Molas E, Barberà MJ, Martín S, Arellano E, Gosch M, Saladie P, Carbonell T, Knobel H, Diez E, Caylà JA (2015). Effectiveness of a pilot partner notification program for new HIV cases in Barcelona, Spain. PLoS One.

[29] Chiou PY, Lin LC, Chen YM, Wu SC, Lew-Ting CY, Yen HW, Chuang P (2015). The effects of early multiple-time PN counseling on newly HIV-diagnosed men who have sex with men in Taiwan. AIDS Behav.

[30] Kahabuka C, Plotkin M, Christensen A, Brown C, Njozi M, Kisendi R, Maokola W, Mlanga E, Lemwayi R, Curran K, Wong V (2017). Addressing the first 90: a highly effective partner notification approach reaches previously undiagnosed sexual partners in Tanzania. AIDS Behav.

[31] Udeagu CCN, Shah D, Shepard CW, Bocour A, Guiterrez R, Begier EM (2012). Impact of a New York City Health Department initiative to expand HIV partner services outside STD clinics. Public Health Rep.

[32] Cherutich P, Golden MR, Wamuti B, Richardson BA, Ásbjörnsdóttir KH, Otieno FA, Ng'ang'a A, Mutiti PM, Macharia P, Sambai B, Dunbar M, Bukusi D, Farquhar C (2017). Assisted partner services for HIV in Kenya: a cluster randomised controlled trial. Lancet HIV.

[33] Rosenberg NE, Mtande TK, Saidi F, Stanley C, Jere E, Paile L, Kumwenda K, Mofolo I, Ng'ambi W, Miller WC, Hoffman I, Hosseinipour M (2015). Recruiting male partners for couple HIV testing and counselling in Malawi's option B+ programme: an unblinded randomised controlled trial. Lancet HIV.

[34] Brown LB, Miller WC, Kamanga G, Nyirenda N, Mmodzi P, Pettifor A, Dominik RC, Kaufman JS, Mapanje C, Martinson F, Cohen MS, Hoffman IF (2011). HIV partner notification is effective and feasible in sub-Saharan Africa: opportunities for HIV treatment and prevention. J Acquir Immune Defic Syndr.

[35] Landis SE, Schoenbach VJ, Weber DJ, Mittal M, Krishan B, Lewis K, Koch GG (1992). Results of a randomized trial of partner notification in cases of HIV infection in North Carolina. N Engl J Med.

[36] Udeagu CCN, Bocour A, Shah S, Ramos Y, Gutierrez R, Shepard CW (2014). Bringing HIV partner services into the age of social media and mobile connectivity. Sex Transm Dis.

[37] Dalal S, Johnson C, Fonner V, Kennedy CE, Siegfried N, Figueroa C, Baggaley R (2017). Improving HIV test uptake and case finding with assisted partner notification services. AIDS.

[38] Shorter MM, Ostermann J, Crump JA, Tribble AC, Itemba DK, Mgonja A, Mtalo A, Bartlett JA, Shao JF, Schimana W, Thielman NM (2009). Characteristics of HIV voluntary counseling and testing clients before and during care and treatment scale-up in Moshi, Tanzania. J Acquir Immune Defic Syndr.

[39] Ostermann J, Reddy EA, Shorter MM, Muiruri C, Mtalo A, Itemba DK, Njau B, Bartlett JA, Crump JA, Thielman NM (2011). Who tests, who doesn't, and why? Uptake of mobile HIV counseling and testing in the Kilimanjaro region of Tanzania. PLoS One.

[40] Program operations. Guidelines for STD prevention. Partner services. Centers for Disease Control and Prevention.

[41] Kerani RP, Fleming M, Golden MR (2013). Acceptability and intention to seek medical care after hypothetical receipt of patient-delivered partner therapy or electronic partner notification postcards among men who have sex with men: the partner's perspective. Sex Transm Dis.

[42] Götz HM, van Rooijen MS, Vriens P, de Coul EO, Hamers M, Heijman T, van den Heuvel F, Koekenbier R, van Leeuwen AP, Voeten HACM (2014). Initial evaluation of use of an online partner notification tool for STI, called 'suggest a test': a cross sectional pilot study. Sex Transm Infect.

[43] Tomnay JE, Pitts MK, Fairley CK (2005). New technology and partner notification--why aren't we using them?. Int J STD AIDS.

[44] Centers for Disease Control and Prevention (CDC) (2004). Using the internet for partner notification of sexually transmitted diseases--Los Angeles County, California, 2003. MMWR Morb Mortal Wkly Rep.

[45] Hightow-Weidman L, Beagle S, Pike E, Kuruc J, Leone P, Mobley V, Foust E, Gay C (2014). "No one's at home and they won't pick up the phone": using the internet and text messaging to enhance partner services in North Carolina. Sex Transm Dis.

[46] Ehlman DC, Jackson M, Saenz G, Novak DS, Kachur R, Heath JT, Furness BW (2010). Evaluation of an innovative internet-based partner notification program for early syphilis case management, Washington, DC, january 2007-june 2008. Sex Transm Dis.

[47] Bernstein KT, Kohn R, Wolf W, Strona F, Fann C, Philip S (2013). O08.1 assessing the added value of internet partner services for syphilis and HIV. Sex Transm Infect.

[48] Klausner JD, Wolf W, Fischer-Ponce L, Zolt I, Katz MH (2000). Tracing a syphilis outbreak through cyberspace. JAMA.

[49] Kachur R, Hall W, Coor A, Kinsey J, Collins D, Strona FV (2018). The use of technology for sexually transmitted disease partner services in the United States: a structured review. Sex Transm Dis.

[50] Pennise M, Inscho R, Herpin K, Owens J, Bedard BA, Weimer AC, Kennedy BS, Younge M (2015). Using smartphone apps in STD interviews to find sexual partners. Public Health Rep.

[51] Vest JR, Valadez AM, Hanner A, Lee JH, Harris PB (2007). Using e-mail to notify pseudonymous e-mail sexual partners. Sex Transm Dis.

[52] Kerani RP, Fleming M, DeYoung B, Golden MR (2011). A randomized, controlled trial of inSPOT and patient-delivered partner therapy for gonorrhea and chlamydial infection among men who have sex with men. Sex Transm Dis.

[53] Rietmeijer CA, Westergaard B, Mickiewicz TA, Richardson D, Ling S, Sapp T, Jordan R, Wilmoth R, Kachur R, McFarlane M (2011). Evaluation of an online partner notification program. Sex Transm Dis.

[54] Paz-Bailey G, Miller W, Shiraishi RW, Jacobson JO, Abimbola TO, Chen SY (2013). Reaching men who have sex with men: a comparison of respondent-driven sampling and time-location sampling in Guatemala City. AIDS Behav.

[55] Semaan S (2010). Time-space sampling and respondent-driven sampling with hard-to-reach populations. Methodol Innov Online.

[56] Johnston LG, Khanam R, Reza M, Khan SI, Banu S, Alam MS, Rahman M, Azim T (2008). The effectiveness of respondent driven sampling for recruiting males who have sex with males in Dhaka, Bangladesh. AIDS Behav.

[57] Hladik W, Barker J, Ssenkusu JM, Opio A, Tappero JW, Hakim A, Serwadda D (2012). HIV infection among men who have sex with men in Kampala, Uganda--a respondent driven sampling survey. PLoS One.

[58] Baldwin R (2014). From regulation to behaviour change: giving nudge the third degree. Mod Law Rev.

[59] Daher J, Vijh R, Linthwaite B, Dave S, Kim J, Dheda K, Peter T, Pai NP (2017). Do digital innovations for HIV and sexually transmitted infections work? Results from a systematic review (1996-2017). BMJ Open.

[60] Conserve DF, Jennings L, Aguiar C, Shin G, Handler L, Maman S (2017). Systematic review of mobile health behavioural interventions to improve uptake of HIV testing for vulnerable and key populations. J Telemed Telecare.

[61] Taylor D, Lunny C, Lolić P, Warje O, Geldman J, Wong T, Gilbert M, Lester R, Ogilvie G (2019). Effectiveness of text messaging interventions on prevention, detection, treatment, and knowledge outcomes for Sexually Transmitted Infections (STIs)/HIV: a systematic review and meta-analysis. Syst Rev.

[62] Paschen-Wolff MM, Restar A, Gandhi AD, Serafino S, Sandfort T (2019). A systematic review of interventions that promote frequent HIV testing. AIDS Behav.

[63] Abaza H, Marschollek M (2017). mHealth application areas and technology combinations*. A comparison of literature from high and low/middle income countries. Methods Inf Med.

[64] Meeker D, Knight TK, Friedberg MW, Linder JA, Goldstein NJ, Fox CR, Rothfeld A, Diaz G, Doctor JN (2014). Nudging guideline-concordant antibiotic prescribing: a randomized clinical trial. JAMA Intern Med.

[65] Thaler RH, Sunstein CR (2009). Nudge: Improving Decisions About Health, Wealth, and Happiness, Revised and Expanded Edition.

[66] Patel MS, Volpp KG, Asch DA (2018). Nudge units to improve the delivery of health care. N Engl J Med.

[67] Bell SFE, Dean JA, Lemoire J, Debattista J, Driver G, Gilks CF, Redmond A, Williams OD (2019). Integrated HIV Self-Testing (HIVST) service delivery in Queensland for policy and service development: study protocol. AIDS Care.

[68] Bourne C, Knight V, Guy R, Wand H, Lu H, McNulty A (2011). Short message service reminder intervention doubles sexually transmitted infection/HIV re-testing rates among men who have sex with men. Sex Transm Infect.

[69] Bourne C, Zablotska I, Williamson A, Calmette Y, Guy R (2012). Promotion and uptake of a new online partner notification and retesting reminder service for gay men. Sex Health.

[70] de Tolly K, Skinner D, Nembaware V, Benjamin P (2012). Investigation into the use of short message services to expand uptake of human immunodeficiency virus testing, and whether content and dosage have impact. Telemed J E Health.

[71] Govender K, Beckett S, Masebo W, Braga C, Zambezi P, Manhique M, George G, Durevall D (2019). Effects of a Short Message Service (SMS) intervention on reduction of HIV risk behaviours and improving HIV testing rates among populations located near roadside wellness clinics: a cluster randomised controlled trial in South Africa, Zimbabwe and Mozambique. AIDS Behav.

[72] Njuguna N, Ngure K, Mugo N, Sambu C, Sianyo C, Gakuo S, Irungu E, Baeten J, Heffron R (2016). The effect of human immunodeficiency virus prevention and reproductive health text messages on human immunodeficiency virus testing among young women in rural Kenya: a pilot study. Sex Transm Dis.

[73] Yao P, Fu R, Rushing SC, Stephens D, Ash JS, Eden KB (2018). Texting 4 sexual health: improving attitudes, intention, and behavior among American Indian and Alaska native youth. Health Promot Pract.

[74] Ybarra ML, Prescott TL, Phillips GL, Bull SS, Parsons JT, Mustanski B (2017). Pilot RCT results of an mHealth HIV prevention program for sexual minority male adolescents. Pediatrics.

[75] Prata N, Weidert K, Soro DR (2021). A mixed-methods study to explore opportunities and challenges with using a mHealth approach to engage men who have sex with men in HIV prevention, treatment and care in Lomé, Togo. Mhealth.

[76] Coleman J, Bohlin KC, Thorson A, Black V, Mechael P, Mangxaba J, Eriksen J (2017). Effectiveness of an SMS-based maternal mHealth intervention to improve clinical outcomes of HIV-positive pregnant women. AIDS Care.

[77] Kassaye SG, Ong'ech J, Sirengo M, Kose J, Matu L, McOdida P, Simiyu R, Syengo T, Muthama D, Machekano R (2016). Cluster-randomized controlled study of SMS text messages for prevention of mother-to-child transmission of HIV in rural Kenya. AIDS Res Treat.

[78] Odeny TA, Bukusi EA, Cohen CR, Yuhas K, Camlin CS, McClelland RS (2014). Texting improves testing: a randomized trial of two-way SMS to increase postpartum prevention of mother-to-child transmission retention and infant HIV testing. AIDS.

[79] Odeny TA, Bukusi EA, Geng EH, Hughes JP, Holmes KK, McClelland RS (2018). Participation in a clinical trial of a text messaging intervention is associated with increased infant HIV testing: a parallel-cohort randomized controlled trial. PLoS One.

[80] George G, Chetty T, Strauss M, Inoti S, Kinyanjui S, Mwai E, Romo ML, Oruko F, Odhiambo JO, Nyaga E, Mantell JE, Govender K, Kelvin EA (2018). Costing analysis of an SMS-based intervention to promote HIV self-testing amongst truckers and sex workers in Kenya. PLoS One.

[81] Kelvin EA, George G, Kinyanjui S, Mwai E, Romo ML, Oruko F, Odhiambo JO, Nyaga EN, Mantell JE, Govender K (2019). Announcing the availability of oral HIV self-test kits via text message to increase HIV testing among hard-to-reach truckers in Kenya: a randomized controlled trial. BMC Public Health.

[82] Kelvin EA, George G, Mwai E, Kinyanjui S, Romo ML, Odhiambo JO, Oruko F, Nyaga E, Govender K, Mantell JE (2019). A randomized controlled trial to increase HIV testing demand among female sex workers in Kenya through announcing the availability of HIV self-testing via text message. AIDS Behav.

[83] National comprehensive guidelines for HIV testing services in Tanzania: third edition. NASHCoP.

[84] Latkin CA, Davey-Rothwell MA, Knowlton AR, Alexander KA, Williams CT, Boodram B (2013). Social network approaches to recruitment, HIV prevention, medical care, and medication adherence. J Acquir Immune Defic Syndr.

[85] Boyer CB, Hightow-Weidman L, Bethel J, Li SX, Henry-Reid L, Futterman D, Maturo D, Straub DM, Howell K, Reid S, Lowe J, Kapogiannis BG, Ellen JM (2013). An assessment of the feasibility and acceptability of a friendship-based social network recruitment strategy to screen at-risk African American and Hispanic/Latina young women for HIV infection. JAMA Pediatr.

[86] (2016). Implementing HIV testing in nonclinical settings: a guide for HIV testing providers. Centers for Disease Control and Prevention.

