# Supplementary figures and images for: Mobile Phone–Based Confidential Social Network Referrals for HIV Testing (CONSORT): Protocol for a Randomized Controlled Trial
Source: JMIR Res Protoc. 2024 May 30;13:e55068. doi: 10.2196/55068 (PMC11176874; doi:10.2196/55068)

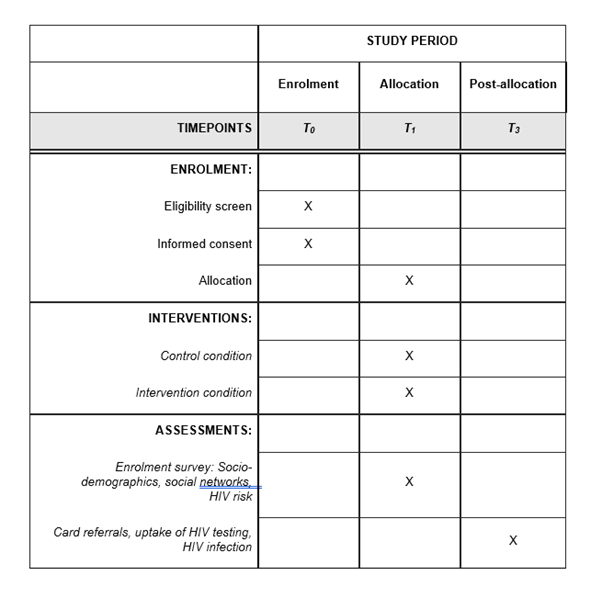

Supplement: Multimedia Appendix 1 [file resprot_v13i1e55068_app1.png]
