# Supplementary material for: Mobile Phone–Based Confidential Social Network Referrals for HIV Testing (CONSORT): Protocol for a Randomized Controlled Trial
Source: JMIR Res Protoc. 2024 May 30;13:e55068. doi: 10.2196/55068 (PMC11176874; doi:10.2196/55068)
Supplement: Multimedia Appendix 3 [file resprot_v13i1e55068_app3.pdf]

1R21TW011667-01A1 Ostermann, Jan

RESUME AND SUMMARY OF DISCUSSION: This resubmitted R21/33 project develops a mobile app and adapts an existing online platform to encourage HIV testing among Tanzanian adults. Only 83 percent of Tanzanians living with HIV are aware of their condition. The untested comprise a higher-risk population. Using traditional public health approaches to reach them is increasingly cost-ineffective. An alternative is to exploit the homophily of social networks to identify the untested and use SMS messages to nudge them toward testing. The research problem is important. Rigorous prior research and preliminary data are reviewed. Capacity building opportunities are described. There are manifold letters of support. The investigators were responsive to prior critiques. Significance was rated high. The excellent investigative team brings ongoing collaboration with NIH funding and complementary expertise in social sciences, HIV control and behavioral and digital health. The University of South Carolina, Duke University and Kilimanjaro Christian Medical Centre/Kilimanjaro Clinical Research Institute provide an optimal research environment. A confidential HIV testing referral system that integrates chain referral, mobile SMS messaging and autonomous open-source system strategies to elicit behavioral change by sending confidential HIV testing invitations to referrals' phone numbers, tracking uptake of HIV testing among referrals, and issuing incentives, is innovative. Formative research, acceptability and efficacy testing, cost-effectiveness and other data analytic methods are clearly outlined. Milestones are measurable. Potential pitfalls and workarounds are discussed. Mixed methods and careful power calculations enhance scientific rigor. In discussion, reviewers were enthusiastic and only minor weaknesses surfaced. There remained concern that sensitive personal information could be compromised if phone messages are seen by others. Whether card referrals are part of standard care is unexplained. On balance, strengths far outweighed weaknesses. The project will significantly impact HIV testing in Tanzania, concluded the panel.

DESCRIPTION (provided by applicant): UNAIDS set for 2030 the ambitious 95-95-95 target: diagnosing 95% of all persons living with HIV (PWH), initiating antiretroviral therapy for 95% of those diagnosed, and achieving viral suppression for 95% of those treated. In Tanzania, a PEPFAR strategy country with an adult HIV prevalence of 4.8%, only 83% of PWH are aware of their status. More than 200,000 undiagnosed PWH need to test in order to reach the "first 95". With the cost-effectiveness of traditional HIV testing approaches declining, we propose to evaluate a novel application of mobile health (mHealth) technology that leverages the ubiquity of mobile phones and the reach of social networks to increase rates of HIV testing, especially among higher-risk individuals. Specifically, we will evaluate the acceptability, efficacy, and cost-effectiveness of automated, confidential, SMS-based HIV testing referrals, as a means of 'nudging' individuals to test. We hypothesize that an automated, confidential referral system, developed and deployed in the Kilimanjaro Region of Tanzania, will be acceptable to both index clients and their referrals and that it will be cost-effective for increasing HIV testing rates. Building on existing relationships with all 25 HIV counseling and testing (HCT) providers in the study area, including 8 HIV care and treatment centers (CTCs), and using an open-source, low-code, and highly versatile mobile phone-based appointment reminder and incentive system (mParis), this study will address the following specific aims: R21 Phase: Aim 1 will conduct qualitative, formative work with (1) HCT providers, (2) HCT clients, (3) PWH who are in care at CTCs, and (4) social network contacts of HCT and CTC clients, to identify desirable provider- and client-side characteristics of an SMS-based HIV testing referral system. Aim 2 will adapt our mParis system to receive phone numbers of HIV testing referrals identified by index clients and autonomously send confidential HIV testing invitations. Aim 3 will pilot-test the Confidential Social Network Referrals for HIV Testing (CONSORT) system to collect preliminary data on the system's acceptability, performance, and potential efficacy. R33 Phase: Aim 4 will evaluate the acceptability and efficacy of CONSORT in a pragmatic randomized controlled trial with 400 HCT clients and 200 HIV-infected CTC patients. Arm 1 participants will be offered confidential SMS referrals and physical invitation cards ("card referrals") to extend to any of

their network contacts. Arm 2 participants will be offered card referrals alone. The primary outcome will be uptake of HIV testing. Secondary outcomes include the number of new HIV diagnoses, and the risk correlation within referral networks. Aim 5 will evaluate the incremental cost-effectiveness of CONSORT+card referrals vs. card referrals alone. The study will provide estimates of the acceptability, efficacy, and cost-effectiveness of an exceptionally low-cost intervention for increasing the uptake of HIV testing. More broadly it will develop analytic and mHealth capacity in Tanzania and inform the development of mobile phone-based chain-referral interventions that can reach key social networks, are transferrable to other technologies, and can be extended beyond HIV testing.

PUBLIC HEALTH RELEVANCE: HIV testing remains the critical first step in achieving the 95-95-95 goals for HIV/AIDS treatment and prevention, yet testing rates are plateauing and more cost-effective strategies are needed to test higher-risk populations. We propose to leverage the ubiquity of mobile phones and the reach of social networks, to evaluate the efficacy and cost-effectiveness of a novel mHealth intervention, Confidential Social Network Referrals for HIV Testing (CONSORT), for 'nudging' high-risk populations to test for HIV. The autonomous, open-source, SMS-based approach can be scaled at minimal cost, is readily extensible to smartphone technologies and social media contexts, and holds significant potential to improve uptake of HIV testing and broadly shift paradigms for engaging social networks to change health-related behaviors.

CRITIQUE 1

Significance: 1
Investigator(s): 1
Innovation: 3
Approach: 1
Environment: 1

Overall Impact: This R21/R33 study seeks to increase HIV screening and initiation of antiretroviral treatment among those screening positive to 95% in Tanzania by leveraging an existing collaboration with researchers in the United States. The R21 phase will a) conduct qualitative research b) adapting an automated system to promote screening and c) pilot test the referral system. The R33 phase will a) conduct a pragmatic randomized trial (n=400) and b) evaluate the cost effectiveness of this approach. Addressing HIV screening and initiation of ART among those screening positive is a significant public health problem in Tanzania which is well suited to a low cost mhealth solution. The proposed research builds upon longstanding collaborations between University of South Carolina and Duke University with investigators in Tanzania. Both the proposed qualitative research and preliminary work as well as the pragmatic trial are well designed. The investigators have effectively addressed concerns raised in the prior review most notably the inclusion of two new investigators (one in US and one in Tanzania) to address concerns regarding privacy and confidentiality of mHealth approach in the context of HIV testing and treatment. The primary limitation of the application is the relatively low to moderate innovation.

1. Significance:

Strengths

- Achieving UNAIDS targets of 95% screening and 95% initiation of ART among PLWHIV is a significant public health mandate that is further emphasized by the prioritization of HIV research at NIH.

- Establishing the effectiveness of a low-cost alternative to promote HIV screening is potentially impactful which is further supported by the ubiquity of cell phone use in Tanzania.
- Tanzania represents an underserved and high-risk population with regard to HIV which is highly stigmatized.
- Chain referral and respondent driven sampling have been shown to impact engagement of hard-to-reach target populations

Weaknesses

- None noted by reviewer.

2. Investigator(s):

Strengths

- The team builds on established and ongoing collaborations between Osterman & Thielman (MPIs)
- MPIs, Osterman & Thielman, provide complementary and integrated expertise
- MPI have long standing and mutually beneficial collaboration with Njau in Tanzania related to HIV testing using mHealth technology.
- Moore & Yamanis provide additional expertise in RDS and social network analysis related to HIV
- van Zwetselaar is an open-source developer and bioinformatician; he developed mPARIS system with Osterman in Tanzania
- The investigators have clarified the PI’s expertise in cost effectiveness research
- Dr. McClimans, an applied medical ethicist, and Ms. Mwangi, Deputy Chair of the Ethics committee at Kilimanjaro Christian Medical were added to address concerns regarding privacy and confidentiality

Weaknesses

- None noted

3. Innovation:

Strengths

- While the sum total of innovation may be moderate, the individual aspects of the approach are not particularly innovative.

Weaknesses

- None of the individual components (i.e., respondent driven sampling, mhealth, cost effectiveness) of the proposal are particularly innovative

4. Approach:

Strengths

- Builds upon an existing mhealth solution (mParis)
- Well outlined qualitative research involving a number of key stakeholder groups (including providers, clients and network contacts)

- Pragmatic trial comparing CONSORT + card referrals vs card referrals is well designed
- Ongoing commitment to building capacity in Tanzania reflected in prior collaborations will continue to be an emphasis in the present

Weaknesses

- None noted

5. Environment:

Strengths

- The School of Public Health at the University of South Carolina and Duke University Global Health Institute and the Center of Health Policy and Inequalities Research provide substantial expertise in carrying out the proposed research
- Existing collaborations at The Kilimanjaro Christian Medical Centre and Clinical Research Institute are robust and will support the completion of the proposed work
- Existing collaborations with HIV Clinics supports viability of recruitment for both the qualitative research and pragmatic trial
- Leverages establishing Community Advisory Board
- Letters of support provide further evidence of commitment to the proposed research in partnering institutions in Tanzania

Weaknesses

- None noted

Milestones (Reference the language in Section V: Additional Review Criteria of PAR 21-303):

Strengths

- Milestones are clearly defined and based on quantitative targets to assess success of R21 phase and transition to R33 phase

Weaknesses

- None noted

Study Timeline:

Strengths

- Timelines for various activities are well defined and overall seem reasonable

Weaknesses

- A disproportionate amount of time may be dedicated to the completion of qualitative work (6 months) and adaptation of existing mhealth platform which is poorly delineated (6 months) in comparison to recruitment timeframe for RCT with n=400. Concerns related to this observations are somewhat minimized by the fact that any extra time would be reinvested in the execution of the RCT and information provided regarding recruitment capacity supports recruitment of sample within the time frame.

Protections for Human Subjects:

Acceptable Risks and/or Adequate Protections

- Appropriate strategies have been outlined to minimize potential risk.
- Concerns regarding privacy and confidentiality have been addressed by the addition of medical ethicists

Data and Safety Monitoring Plan (Applicable for Clinical Trials Only):

Acceptable

- DSMP well outlined

Inclusion Plans:

- Sex/Gender: Distribution justified scientifically
- Race/Ethnicity: Distribution justified scientifically
- For NIH-Defined Phase III trials, Plans for valid design and analysis: Scientifically acceptable
- Inclusion/Exclusion Based on Age: Distribution justified scientifically
- key aspects of inclusion/exclusion were addressed by applicant including consideration of younger participants (16 years of age) and efforts to insure 50:50 balance in gender

Vertebrate Animals:

NO, animal welfare concerns or incomplete

Biohazards:

Not Applicable (No Biohazards)

Resubmission:

- Clarified available expertise in cost effectiveness research
- Clarified continuing commitment to capacity building in Tanzania
- Added two medical ethicist (one in US and one in Tanzania) to address concerns regarding privacy and confidentiality

Applications from Foreign Organizations:

Justified

- Tanzania is significantly impacted by HIV which is highly stigmitized
- Highly productive, long standing collaboration between US and Tanzanian Institutions and Investigators

Resource Sharing Plans:

Acceptable

- Investigators provide additional information supporting ongoing commitment to capacity building

Budget and Period of Support:

Recommend as Requested

CRITIQUE 2

Significance: 1
Investigator(s): 1
Innovation: 1
Approach: 2
Environment: 1

Overall Impact: This resubmission from the Univ of South Carolina, Duke Univ, and Kilimanjaro Christian Medical Univ seeks to increase rates of HIV testing among high-risk individuals. mParis, a mobile phone based appointment reminder and incentive system (open-source, low code), will be updated to allow confidential distribution of nudges to obtain HIV testing to the close contacts of an index patient, who is undergoing HIV testing. The R21 phase will conduct qualitative work with multiple stakeholders to identify provider-and client-side design needs, build the CONSORT system, and pilot test the intervention. The R33 phase will evaluate acceptability and efficacy in a pragmatic RCT with 400 HCT clients and 200 HIV infected CTC patients, as well as a cost-effectiveness evaluation. The investigators have strong expertise in all methods and subject areas covered by the application, and they have added 2 additional personnel to help with the ethical issues around confidential contact of social networks. Although the underlying premise of this work is high-risk, I think it is important to pursue the exploratory and feasibility work to test out whether it could contribute to increased HIV testing rates. The resubmission is very responsive to the prior reviews, and the approach is strong.

1. Significance:

Strengths

- The investigators make the case that an important step is to target, at low cost, large numbers of individuals who are at above-average risk of HIV infection. Social network members share similar characteristics, including HIV risk profiles, and therefore are worth examining – this is novel because members of hidden populations are best accessed through their peers
- Prior research has focused primarily on ‘visible’ and ‘directly accessible’ populations
- Strong scientific rationale and support for the sustainability of this program

Weaknesses

- A strong rationale is provided for how this approach will reach a wider group of high-risk individuals, but lacks rationale for whether social network contacts will respond to these messages.

2. Investigator(s):

Strengths

- PI Ostermann has extensive experience in HIV research, particularly decision making around health and preventive behaviors, testing interventions, and cost-effectiveness analysis.
- Dr. Njau has expertise in qualitative and survey research

- Dr. Thielman has decades of experience in HIV research in Tanzania, particularly around testing decision-making
- Dr. Mwangi is a behavioral scientist with expertise in qualitative and health education
- Dr. Yamanis is a doctoral student who studies social network analysis and ethical issues in HIV prevention research. Prior research has included diverse sampling approaches to reach populations at high risk of HIV.
- The research team recognizes the ethical issues around confidential HIV testing invitations, and has an ethicist in the key personnel.
- Researchers have collaborated successfully in prior work

Weaknesses

- None

3. Innovation:

Strengths

- The integration of mhealth and social networks is highly novel
- Leveraging social networks and technologies for nudging is readily extensible to other areas of public health (e.g., vaccine hesitancy) – this is relevant globally
- One of the main strengths of this application is its innovation – it is high-risk, but likely worth evaluating the acceptability and feasibility of this approach.

Weaknesses

- None

4. Approach:

Strengths

- Strong technical description of mParis and CONSORT is provided – this conveys clear expertise and understanding of how technical aspects influence design of the studies.
- Prelim data on moderate acceptability is provided – both for willingness to share contact info and respond to anonymous texts
- Both index clients and referrals are included in the formative research in Aim 1
- Possible content of SMS messages is clearly provided – and how participants could block, opt out, or not store the messages are clearly described, and it is good to have participants experience what it would feel like to receive one of the CONSORT messages
- The authors recognize that the efficacy of CONSORT hinges on its acceptability to index clients and referrals, and alternate strategies are proposed.
- The design of the RCT and cost-effectiveness analysis are rigorously described. The use of an arm with card referrals is a good choice. Acceptability and efficacy outcome measures are clear.

Weaknesses

- In the acceptability/feasibility study, it would be important to examine whether partner might see message and risk of violence against the recipient

- In Aim 3, will the referral clients be told who referred them? If yes, how will a breach in confidentiality influence index participant willingness to take part in the study? If not, how will the research team respond if the referral identifies the index patient?
- In Aim 4 – are card referrals the standard of care?

5. Environment:

Strengths

- Duke Univ has the Global Health Institute (DGHI), the Center for Health Policy and Inequalities Research (CHPIR), and the Duke Center for AIDS Research (CFAR)
- Kilimanjaro Christian Medical Centre (KCMC) has strong research infrastructure and a CAB, and existing software (mParis) upon which to build the current intervention
- Builds upon existing relationships with counseling and testing centers in the study area

Weaknesses

- None

Milestones (Reference the language in Section V: Additional Review Criteria of PAR 21-303):

Strengths

- Well described

Weaknesses

- None

Study Timeline:

Strengths

- Well described, feasible

Weaknesses

- None

Protections for Human Subjects:

Acceptable Risks and/or Adequate Protections

- No concerns

Data and Safety Monitoring Plan (Applicable for Clinical Trials Only):

Acceptable

- No concerns

Inclusion Plans:

- Sex/Gender: Distribution justified scientifically
- Race/Ethnicity: Distribution justified scientifically
- For NIH-Defined Phase III trials, Plans for valid design and analysis: Not applicable

- Inclusion/Exclusion Based on Age: Distribution justified scientifically
- No concerns

Vertebrate Animals:
Not Applicable (No Vertebrate Animals)

Biohazards:
Not Applicable (No Biohazards)

Resubmission:
- Very responsive to prior critiques

Applications from Foreign Organizations:
Justified
- Clear rationale is presented

Budget and Period of Support:
Recommend as Requested

CRITIQUE 3

Significance: 1
Investigator(s): 1
Innovation: 2
Approach: 2
Environment: 1

Overall Impact: Investigators will evaluate the acceptability, efficacy, and cost-effectiveness of Confidential Social Network Referrals for HIV Testing (CONSORT), a system that uses SMS-based referrals to encourage HIV testing in Tanzania building on a previously-developed platform: mobile phone-based appointment reminder and incentive system (mParis). In Phase 1 (R21) they will use qualitative methods to conduct a formative assessment with HIV testing providers, patients, PWH in care, and members of patients' social networks to identify desirable characteristics of the SMS system; then adapt their mParis system to receive phone numbers from index clients of those who might test, to whom they will in turn send confidential HIV testing invitations; and finally pilot the system, collecting data regarding acceptability, performance, and potential efficacy. In Phase 2 (R33) they will evaluate the acceptability and efficacy of their system for leveraging social networks to refer for HIV testing via an RCT with 400 testing clients and 200 PWH. They will also evaluate the incremental cost-effectiveness of their system as compared to a referral process that relies only on physical invitation cards (with no SMS referrals.) In addition to providing data regarding the acceptability, efficacy, and cost-effectiveness of this approach, investigators aim to develop analytic and mHealth capacity in Tanzania and to inform the development of phone-based chain referral interventions that can reach social networks and will be transferrable to other technologies and public health foci. The investigator

team is quite strong and builds on long-standing collaborations with each other and with local HIV Counseling and Testing/Care Centers. Potential impact is high.

1. Significance:

Strengths

- UNAIDS HIV goals for diagnosing 95% of PWH remain a priority for LMIC including Tanzania, where investigators note 200,000 in that region remain untested.
- Tanzania has high HIV prevalence but also concentrated epidemics among MSM, female sex workers, and injection drug users. These populations might not be reached through traditional case-finding efforts.
- Social and sexual networks play important roles in HIV testing and care decision-making, and the ubiquity of mobile phones in many high-prevalence areas including in LMIC present the opportunity to leverage those networks via a low-cost approach.
- Investigators note the study will increase digital health and analytic capacity/expertise in Tanzania, providing infrastructure that will be able to be leveraged in future studies.
- If found acceptable, effective, and low-cost, this confidential approach to chain-referrals building on open source architecture could be extended to many other settings, populations, and health issues.
- The approach builds on the premises that PWH’s sexual partners and other members of their social networks share risk profiles, and that chain referrals can effectively reach those who might otherwise remain unreached.

Weaknesses

- None noted.

2. Investigator(s):

Strengths

- Investigators demonstrate a strong foundation of collaboration in Tanzania over the past 15 years, supported by multiple NIH-funded studies. In addition to the research infrastructure and relationships this study can build upon, they state they have a thorough understanding of local context and have collaborated with all 25 HIV counseling and testing sites in the region, which includes 8 care and treatment centers.
- Investigators previously developed a mHealth system, which this study will build upon. The proposal includes an experienced programming consultant who will previously collaborated with the Contact PI on the development of mParis.
- The MPIs have previously collaborated on multiple funded studies.
- Roles of MPIs are fully described.
- Two new in-country co-investigators have been added to this resubmission, including a medical ethicist and deputy chair of the ethics committee of the Kilimanjaro Christian Medical Centre. They will ensure ethical aspects of the study are appropriately considered, especially regarding confidentiality of HIV testing message.

Weaknesses

- None noted.

3. Innovation:

Strengths

- The study will provide needed information about the utility of using mHealth-based approaches in confidential partner notification strategies, which to date has been unevaluated.
- CONSORT is potentially a very low-cost and largely autonomous implementation approach.
- Nudging approaches to increase HIV testing remain rare, and few studies have evaluated SMS interventions to encourage HIV testing. Building SMS nudging on the concept of partner notification is particularly innovative.
- Pairing open-source autonomous SMS technology, referrals based on social networks (with whom index clients are likely to have similar risk profiles), and nudging to encourage HIV testing are in combination quite innovative.

Weaknesses

- None noted.

4. Approach:

Strengths

- Comparing performance of the Confidential Social Network Referrals for HIV Testing (CONSORT) using SMS-based testing invitations to that of card referrals-only will provide useful data regarding the value added as well as comparative cost-effectiveness.
- Study methods build on pilot data showing that index clients would be likely to invite contacts for HIV testing, and that 1000 index clients would yield 638 HIV tests ad a cost of $0.34 per HIV diagnoses.
- Aim 1 will use focus group discussion (FGD) to elucidate client-side characteristics (messaging, ethical considerations, potential adverse events) of CONSORT. Half of participants will be recruited as index clients, with the other half being recruited as referrals invited or confidentially referred by the index clients, which provides interesting tests of the approach. Qualitative methods are appropriately theory-driven (Planned Behavior, Social Cognitive, Game Theory.)
- Aim 1 also includes a solid plan for piloting the CONSORT intervention with 100 participants. Acceptability, performance, and potential efficacy measures are appropriate and likely to provide data needed to transition to Phase 2.
- Aim 2 focuses on adapting mParis to provide a confidential, autonomous means of referral to testing. Maintaining confidentiality of the index clients is a critical strength, and the fact that the system is autonomous further protects confidentiality and reduces costs.
- Phase 2 incorporates an RCT in which participants (n=500) will be randomized to CONSORT plus card referrals or to card referrals alone. This is a stronger test of testing uptake than CONSORT-only as it captures substitution effects.
- The planned cost-effectiveness analysis will provide valuable information regarding the potential gain and overall significance of CONSORT.
- Methods for opting out of text messages are described.

Weaknesses

- Pilot data reflecting hypothetical feasibility and acceptability of CONSORT was collected mainly from females, with low numbers of male participants (only 32 of 145.)
- In Aim 1, interviews will be conducted with only 1 provider from each of the 25 HCT sites. There is no description of provider recruitment, and with only 1 per site, there is no assurance of representativeness.
- There is an intent to build mHealth capacity in Tanzania, but this plan has not yet been developed.

5. Environment:

Strengths

- USC, Duke (where the contact PI is also an adjunct), and Kilimanjaro Christian Medical Centre are excellent environments for this research; these institutions have a history of successful collaboration in the target region.
- Strong letters of support from local collaborators are included in the application.

Weaknesses

- None noted.

Milestones (Reference the language in Section V: Additional Review Criteria of PAR 21-303):

Strengths

- Transition milestones are well-described and include measures for acceptability, efficacy of reach, and efficacy for bringing referrals to test. These measures rely on successful consecutively sent messages (i.e., 16 of the 20 most recent messages sent are confirmed to have been delivered), a more meaningful test of implementation than just a proportion of successful messages received.
- Milestones are conservative, and investigators indicate that in the worst-case scenario with only the minimum threshold met for each milestone, there will be sufficient data to transition to phase 2.

Weaknesses

- None noted.

Study Timeline:

Strengths

- The timeline appears feasible, with the first year dedicated to study start up and measures for informing CONSORT.

Weaknesses

- None noted.

Protections for Human Subjects:

Acceptable Risks and/or Adequate Protections

- No concerns noted.

- There is a plan to explore a waiver of parental consent since Tanzanian national policy indicates adolescents under 18 who are sexually active have the right to access reproductive health services without parental consent.

Data and Safety Monitoring Plan (Applicable for Clinical Trials Only):

Acceptable

- No concerns noted.

Inclusion Plans:

- Sex/Gender: Distribution justified scientifically
- Race/Ethnicity: Distribution justified scientifically
- For NIH-Defined Phase III trials, Plans for valid design and analysis: Scientifically acceptable
- Inclusion/Exclusion Based on Age: Distribution justified scientifically
- No concerns noted.

Vertebrate Animals:

Not Applicable (No Vertebrate Animals)

Biohazards:

Not Applicable (No Biohazards)

Resubmission:

- The responses to previous reviewers' concerns is comprehensive. The experience of the MPI who will conduct the CEA is now fully described, which was a concern noted by previous reviewers.

Applications from Foreign Organizations:

Justified

- The plan to collaborate with investigators from and organizations in Tanzania is excellent, particulaty given the local burden of HIV and their planned roles providing input on local context and review of ethical approaches.

Resource Sharing Plans:

Acceptable

- No concerns noted.

Budget and Period of Support:

Recommend as Requested

- No concerns noted.

THE FOLLOWING SECTIONS WERE PREPARED BY THE SCIENTIFIC REVIEW OFFICER TO SUMMARIZE THE OUTCOME OF DISCUSSIONS OF THE REVIEW COMMITTEE, OR REVIEWERS' WRITTEN CRITIQUES, ON THE FOLLOWING ISSUES:

PROTECTION OF HUMAN SUBJECTS: ACCEPTABLE

INCLUSION OF WOMEN PLAN: ACCEPTABLE

INCLUSION OF MINORITIES PLAN: ACCEPTABLE

INCLUSION ACROSS THE LIFESPAN: ACCEPTABLE

COMMITTEE BUDGET RECOMMENDATIONS: The budget was recommended as requested.

Footnotes for 1 R21 TW011667-01A1; PI Name: Ostermann, Jan

Ad hoc or special section application percentiled against "Total CSR" base.

NIH has modified its policy regarding the receipt of resubmissions (amended applications).See Guide Notice NOT-OD-18-197 at https://grants.nih.gov/grants/guide/notice-files/NOT-OD-18-197.html. The impact/priority score is calculated after discussion of an application by averaging the overall scores (1-9) given by all voting reviewers on the committee and multiplying by 10. The criterion scores are submitted prior to the meeting by the individual reviewers assigned to an application, and are not discussed specifically at the review meeting or calculated into the overall impact score. Some applications also receive a percentile ranking. For details on the review process, see http://grants.nih.gov/grants/peer_review_process.htm#scoring.
